# Cognitive Behavior Therapy for Patients With Cancer

**Published:** 2015-01-01

**Authors:** Sheena Daniels

**Affiliations:** Florida Agricultural & Mechanical University, Tallahassee, Florida

Cancer is the second most common cause of death in the United States. It is estimated that there were 1,665,540 new cases diagnosed in 2014 (American Cancer Society [ACS], 2014). The mainstay treatment options for various cancers include surgery, radiation, chemotherapy, and hormonal therapies or some combination. In addition to the effects cancer itself may have on the patient, its treatment often brings about adverse effects such as fatigue, insomnia, pain, and depression (ACS, 2014). These side effects can have an impact on the patient’s physical condition as well as on his or her quality of life.

Because of the multiple effects cancer can have on a patient’s life, it is important for oncology advanced practitioners (APs) to include both pharmacologic and nonpharmacologic management methods in the plan of care. One intervention that has been recognized as beneficial is cognitive behavior therapy, or CBT (Brothers, Yang, Strunk, & Andersen, 2011; Greer, 2008; Lee, Lim, Yoo, & Kim, 2011; Tatrow & Montgomery, 2006). This article will describe the use of CBT as an intervention for patients with cancer and the positive impact it may have on quality of life.

## WHAT IS CBT?

Cognitive behavior therapy is a psychotherapeutic approach that emphasizes the significance of how our thinking affects the way we feel. It has historically been used for psychological disorders yet is now being explored for a number of different problems, including pain associated with various oncologic and hematologic disorders (Anie & Green, 2012; Tatrow & Montgomery, 2006).

Cognitive behavior therapy is built on the foundation that it is difficult to change our emotions directly. The aim of CBT is to change emotions by first changing thoughts and behaviors (Cully & Teten, 2008). Offered in 30- to 60-minute increments, CBT is targeted to change the perceptions of how and what patients think based on the basic principle that says how a person thinks has a tremendous effect on his or her emotions and behavior (Mustaffa, Musa, Abu, & Yusof, 2012). The patient works with a CBT practitioner to develop skills to recognize, counteract, and manage problematic thoughts and beliefs (Aschim et al., 2011).

If resources are available, APs can refer patients to licensed cognitive behavior therapists; many counselors have extensive training in this technique, which would be a benefit to both the AP and the patient. However, if these resources are not accessible, APs are in a perfect position to offer CBT to their patients with minimal training.

Cognitive behavior therapy sessions can be divided into stages that start with gathering important information about the patient (and what concerns the patient has) and end with a final stage that helps the patient integrate what was learned in the sessions to help them cope with the concerns associated with living with cancer (Mustaffa, Musa, Abu, & Yusof, 2012).

The first stage generally focuses on identifying the problem. For example, the AP would ask the patient questions: "What made you come here today? What is the biggest challenge you are facing?" Gathering this information helps the AP identify which approach and technique would benefit the patient most. For instance, Susan has stated that she has been feeling nauseated from the side effects of chemotherapy. The AP would then discuss the potential techniques that could be used, such as cognitive restructuring. Using this particular technique, cognitive restructuring, would include asking Susan to identify her negative thoughts and the impact those negative thoughts would likely have during her chemotherapy treatments. The AP would then ask her, "What can be changed about the situation? Is there anything you can change about how you think that could possibly make you feel better?" This open-ended question will lead Susan into exploring different perspectives and possibly changing her feelings or thought processes. The AP then might ask Susan to start thinking and journaling positive thoughts and to practice this positivity in her daily life and during chemotherapy treatments (Mustaffa et al., 2012).

## TRIALS IN CBT

A number of studies have been conducted indicating that CBT is a beneficial therapy option that can be utilized for various cancer patients and for a range of symptoms (Brothers et al., 2011; Greer, 2008; Lee et al., 2011; Tatrow & Montgomery, 2006). Cognitive behavior therapies may include cognitive restructuring, relaxation, skills training, and visual imagery, among other modalities. Lee et al. (2011) conducted an exploratory study in patients with breast cancer who were undergoing radiotherapy and were experiencing side effects, including fatigue and decreased quality of life. After a 6-week nurse-led CBT intervention, Lee et al. found that the participants were able to better control their fatigue levels and had significantly higher quality of life than the participants in the control group.

Brothers et al. (2011) conducted a study with 36 cancer survivors who were diagnosed with major depressive disorder to determine the effectiveness of biobehavioral and cognitive behavior interventions. They found that patients receiving CBT reported improvements in their mental health, depression, and fatigue posttreatment.

Distress and pain are other common side effects associated with cancer and its treatment. Cognitive behavior therapy has been linked to alleviating both of these concerns (Greer, 2008; Tatrow & Montgomery, 2006). A meta-analysis conducted by Tatrow and Montgomery (2006) studied the use of CBT for distress and pain in breast cancer patients. That study revealed that 69% of the patients who participated in the treatment group reported less pain and less distress. Greer (2008) observed that fostering positive environments and building rapport with patients are essential and can aid in the effectiveness of CBT in reducing helplessness and hopelessness.

## BARRIERS TO IMPLEMENTATION

Barriers to APs offering CBT to their patients who have been identified as potential candidates include distractions within the practice, lack of time, and various interruptions. Barriers to patients accepting or actually using CBT include having a preference for pharmacotherapy and lack of interest and/or motivation (Wiebe & Griever, 2005). Advanced practitioners can try to overcome some of these barriers by starting small: incorporate a condensed version of CBT that fits your schedule, give the patient "homework," and provide feedback on the return visit (Wiebe & Griever, 2005). Completing homework and receiving feedback can make the patient feel like a full participant in his or her health care.

## HOW CAN APs IMPLEMENT CBT?

Oncology APs can implement CBT into their practice in a number of ways. Studies have shown that incorporating CBT into regularly scheduled medical visits can be a practical way to integrate it into patient care (Keefe, Abernathy, & Campbell, 2005). The use of guided self-help CBT through books, manuals, and handouts has been studied. It has been found to be more effective in improving mood for the treatment of depression than usual treatment alone (Williams et al., 2005). A review on the effectiveness of CBT in primary health care suggested that primary care practitioners are in a good position to offer this type of therapy to patients without extensive specialized training; they found that it is an effective therapy for anxiety and depression and can be delivered in primary care (Høifødt, Strøm, Kolstrop, Eisemann, & Waterloo, 2011). Additionally, the review described other methods of delivery, including Internet/computer, face-to-face, and self-help materials. This is consistent with another study that found educated partner–guided CBT was beneficial in helping increase self-efficacy and reduce pain (Keefe et al., 2005).

There are informal and formal ways for APs to learn how to implement CBT. There are a number of CBT manuals available for the AP who has no formal CBT training. These manuals are designed to give a brief therapy model to assist in rapid training (Cully & Teten, 2008; Rosselló & Bernal, 2007). Formal training is available through the Beck Institute for CBT, which offers both individual and group training on-site as well as consultations for your facility.

## CONCLUSION

The therapies we use for cancer treatment often affect the patient mentally as well as physically, causing distress and symptoms that affect quality of life. Oncology APs can integrate CBT in a variety of ways and allow nonpharmacologic treatments to aid in the symptom management that comes with a cancer diagnosis. Implementing CBT into practice may appear challenging at first, but efforts to help this unique population in achieving better all-around health are efforts well spent.
